# *Caenorhabditis elegans* as an Infection Model for Pathogenic Mold and Dimorphic Fungi: Applications and Challenges

**DOI:** 10.3389/fcimb.2021.751947

**Published:** 2021-10-15

**Authors:** Chukwuemeka Samson Ahamefule, Blessing C. Ezeuduji, James C. Ogbonna, Anene N. Moneke, Anthony C. Ike, Cheng Jin, Bin Wang, Wenxia Fang

**Affiliations:** ^1^ National Engineering Research Center for Non-Food Biorefinery, Guangxi Academy of Sciences, Nanning, China; ^2^ College of Life Science and Technology, Guangxi University, Nanning, China; ^3^ Department of Microbiology, University of Nigeria, Nsukka, Nigeria; ^4^ Department of Microbiology, University of Jos, Jos, Nigeria; ^5^ State Key Laboratory of Non-Food Biomass and Enzyme Technology, Guangxi Academy of Sciences, Nanning, China

**Keywords:** *Caenorhabditis elegans*, dimorphic fungi, filamentous fungi, *in vivo* model, pathogenicity, high-throughput screening

## Abstract

The threat burden from pathogenic fungi is universal and increasing with alarming high mortality and morbidity rates from invasive fungal infections. Understanding the virulence factors of these fungi, screening effective antifungal agents and exploring appropriate treatment approaches in *in vivo* modeling organisms are vital research projects for controlling mycoses. *Caenorhabditis elegans* has been proven to be a valuable tool in studies of most clinically relevant dimorphic fungi, helping to identify a number of virulence factors and immune-regulators and screen effective antifungal agents without cytotoxic effects. However, little has been achieved and reported with regard to pathogenic filamentous fungi (molds) in the nematode model. In this review, we have summarized the enormous breakthrough of applying a *C. elegans* infection model for dimorphic fungi studies and the very few reports for filamentous fungi. We have also identified and discussed the challenges in *C. elegans-*mold modeling applications as well as the possible approaches to conquer these challenges from our practical knowledge in *C. elegans-Aspergillus fumigatus* model.

## Introduction

Pathogenic fungi pose an enormous global threat to humanity, leading to millions of deaths and substantial financial losses annually (Fisher et al., 2012; Rhodes, 2019). Morbidity and mortality rates from opportunistic fungal pathogens, such as *Candida albicans*, *Aspergillus fumigatus*, and *Cryptococcus neoformans*, have been increasing for some years, especially in immunocompromised patients (Pal, 2017; Linder et al., 2019; de Sousa-Neto et al., 2020). Addressing the pathogenesis of these fungal pathogens and finding controllable strategies are crucial and urgent. To tackle this threat, model organisms are required to conduct research focusing on the identification of virulence factors, screening of effective antifungal agents, and exploring appropriate treatment approaches.

Several model organisms have been adopted for studying of dimorphic and filamentous pathogenic fungi, including invertebrate models such as *Drosophila melanogaster* (Lamaris et al., 2008; Regulin and Kempken, 2018; Sampaio et al., 2018; Wurster et al., 2019), *Galleria mellonella* (Gomez-Lopez et al., 2014; Long et al., 2018; Silva et al., 2018; Staniszewska et al., 2020), *Bombyx mori* (Matsumoto et al., 2013; Uchida et al., 2016; Nakamura et al., 2017; Matsumoto and Sekimizu, 2019), *Caenorhabditis elegans* (Okoli and Bignell, 2015; Song et al., 2019; Wong et al., 2019; Ahamefule et al., 2020a), and vertebrate models such as mice (Fakhim et al., 2018; Skalski et al., 2018; Wang et al., 2018; Mueller et al., 2019), guinea pigs (Vallor et al., 2008; Nadăş et al., 2013; Garvey et al., 2015), and zebrafish (Chen et al., 2015; Knox et al., 2017; Koch et al., 2019; Kulatunga et al., 2019).

*C. elegans* is a microscopic multicellular nematode that lives freely in soil (Muhammed et al., 2012; Kim et al., 2017). Advantages, such as short life cycle, physiological simplicity, transparent body, complete sequenced genome, mature genetic manipulation system, and no requirement for ethical license, have greatly encouraged the wide adoption of this nematode as a model organism in scientific research with assorted applications across several research fields (Okoli et al., 2009; Ballestriero et al., 2010; Huang et al., 2014; Jiang and Wang, 2018). Some of these applications have been established for decades now whereas others are still in their nascent stages undergoing several studies. Nematode infection by the natural nematophagous obligate filamentous fungus *Drechmeria coniospora* is a common incidence in nature. *C. elegans* is usually applied for studying the innate immunity of nematodes to this fungus (Engelmann et al., 2011; Couillault et al., 2012; Zugasti et al., 2016). This nematode model has also been explored as an *in vivo* model for studying infections of human pathogenic filamentous fungi (Okoli and Bignell, 2015; Ahamefule et al., 2020a).

Application of the nematode model for dimorphic pathogenic fungi studies has resulted in numerous publications whereas only a few publications thus far have been recorded for human filamentous pathogenic fungi studies, such as *A. fumigatus* (Okoli and Bignell, 2015; Ahamefule et al., 2020a; Eldesouky et al., 2020a). Here, we have extensively portrayed *C. elegans-*dimorphic fungi (in particular *Candida* spp.) infection models for determining virulence factors (reported within the last decade) and evaluated the effectiveness of anticandidal agents, including drugs, bioactive compounds, and live biotherapeutic products (reported within the last 5 years). The practical challenges constraining the applications of the *C. elegans* model for filamentous fungi are elaborated, and possible solutions are raised for future improvement.

## Application of *C. elegans* for Dimorphic Fungi Studies

*C. elegans* has been extensively used for studying several dimorphic fungi of clinical relevance. The most devastating and pathogenic dimorphic fungus that has been adequately explored with this nematode model is *Candida albicans* (Hans et al., 2019a; Hans et al., 2019b; Song et al., 2019; Venkata et al., 2020) and a few other non-*albicans* species such as *C. tropicalis* (Brilhante et al., 2016; Feistel et al., 2019; Pedroso et al., 2019), *C. krusei* (De Aguiar Cordeiro et al., 2018; Kunyeit et al., 2019), and *C. auris* (Eldesouky et al., 2018a; Mohammad et al., 2019). Another important clinical dimorphic fungus, *Taloromyces* (*Penicillium*) *marneffei*, has also been studied in a *C. elegans* model for both virulence tests and antifungal agent efficacy evaluations (Huang et al., 2014; Sangkanu et al., 2021).

Virulence factors of *C. albicans* such as genes involved in hyphal filamentation and biofilm formation (Romanowski et al., 2012; Sun et al., 2015; Holt et al., 2017), intestinal adhesion and colonization (Rane et al., 2014a; Muthamil et al., 2018; Priya and Pandian, 2020), important virulence enzymes (Ortega-Riveros et al., 2017; Song et al., 2019), transcription factors (Jain et al., 2013; Hans et al., 2019a), and environmental and nutrient factors (Hammond et al., 2013; Lopes et al., 2018; Hans et al., 2019b; Wong et al., 2019) have been identified in a *C. elegans* model to strengthen our understanding of the *in vivo* pathogenesis of this important fungal pathogen (**Table 1**). The virulence traits of some other non-*albicans* species (both dimorphic and nondimorphic) have also been investigated with this nematode model (**Table 1**). Similarly, virulence factors such as pigmentation and hyphal filamentation have been demonstrated to be critical pathogenic features of *T. marneffei* in a *C. elegans* infection model (Huang et al., 2014; Sangkanu et al., 2021). *C. elegans glp-4; sek-1* worms have mostly been used in these studies (aside from the wild-type strain, N2) because of their inability to produce progeny at 25°C due to the *glp-4* mutation and their susceptibility to pathogens due to *sek-1* mutation, thus making the worms immunocompromised for infection by opportunistic human fungi (Huang et al., 2014; Okoli and Bignell, 2015; Ahamefule et al., 2020a)

**Table 1:** Application of *C. elegans* in determining/confirming *in vivo* virulence of *Candida* spp.

*Candida* spp.	*C. elegans* strain used	Identified virulence factors/conditions	Effect on host	References
*C. albicans*	N2 Bristol (wild type) and *sek-1Δ* worms	Transcription factor *CAS5* Kinase *CEK1* Transcription factor *RIM101*	Avirulence or attenuated virulence of pathogen in host.	Feistel et al. (2019)
*C. albicans*, *C. dubliniensis*, *C. tropicalis*, *C. parapsilosis*	N2 Bristol (wild type) and *sek-1Δ* worms	Screen diverse pathogen strain backgrounds and species	*C. albicans*, *C. tropicalis*, and *C. dubliniensis* gave the most virulent effect on healthy nematode populations while *C. parapsillosis*, *C. tropicalis*, and *C. albicans* were the most virulent on immunocompromised worms	Feistel et al. (2019)
*C. albicans*	*glp-4; sek-1* adult worms	Alcohol dehydrogenase 1 (*ADH1*)	Significant (*p* < 0.05) increase in survival time of worms infected by *ADHI* mutant strain (adh1Δ/Δ) compared with the wild-type and reconstituted strains	Song et al., 2019
*C. albicans*	N2 L4-young adult worms	Filamentation and virulence induced by phosphate conditions	Strain ICU1 caused mortality in worms in a phosphate-dependent manner while ICU12 caused mortality both in low and high phosphate conditions albeit consistent with degree of filamentation. Worms generally displayed an avoidance behavior on *C. albicans* grown in low phosphate medium	Romanowski et al. (2012)
*C. albicans*	N2 L4 worms	Prevacuolar protein sorting gene (*VPS4*) needed for extracellular secretion of aspartyl proteases	Attenuated virulence by *vps4Δ* (66 h median survival) compared with wild type, DAY185 (42 h), and reintegrant strains (45 h)	Rane et al. (2014a)
*C. albicans*	N2 Bristol larval and adult worms	Effects of microgravity on virulence	Reduced virulence in both larval and adult worms in spaceflight; reduced virulence in only larval and not adult worms in clinorotation all compared with static ground controls	Hammond et al. (2013)
*C. albicans*	N2 Bristol L4 worms	Hypoxia (1% oxygen)	Enhanced significant virulence (*p <* 0.001) leading to more than 80% worm mortality compared with controls	Lopes et al. (2018)
*C. albicans*	AU37 (*glp-4; sek-1*) worms	Limiting phospholipid synthesis	Approximately 23%–38% virulence reduction in mutant strains (*LRO1*, *CHO1*, and *LPT1*) compared with control	Wong et al. (2019)
*C. albicans*	*glp-4; sek-1* adult worms	Transcription coactivator *SPT20*	Attenuated virulence. Absence of hyphae filamentation in worms infected by mutant strains as against visible hyphae protrusion recorded in approximately half of dead worms infected by both wild-type and reintegrated strains at 48 h	Tan et al. (2014)
*C. albicans*	N2 young adult worms	[helix–loop–helix/leucine zipper (bHLH/Zip)] transcription factor *CaRTG3*	Significant increased survival rate (*p* < 0.05) of worms infected with *rtg3* mutant strain (43.3%) compared with the wild-type (6.6%) and revertant (10%) strains	Hans et al. (2019a)
*C. albicans + Staphylococcus epidermidis*	*glp-4; sek-1* L3 and L4 worms	Biofilm and hyphal filamentation	Significantly reduced survival rate (*p* < 0.05) of coinfected worms (47%) compared with single infection by *C. albicans* hyphae (63%) and yeasts (81.5%) phenotypes	Holt et al. (2017)
*C. albicans*	*glp-4; sek-1* adult worms	Iron-sulfur subunit of succinate dehydrogenase *SDH2*	More than 85% mortality of worms infected with wild-type and reintegrated strains (all with visible hyphae) compared with 0% mortality and total absence of hyphae in worms infected with mutant (*sdh2Δ/Δ*) at 120 h	Bi et al. (2018)
*C. albicans*	N2 L4 worms	Proton pump V-ATPase	The tetR-VMA2 mutant was avirulent	Rane et al. (2014b)
*C. albicans*	*glp-4; sek-1* L4 worms	Molecular chaperone Hsp104	Significant increase in survival rate (*p* < 0.05) in Hsp104 homozygous mutant strain (17.2%) relative to heterozygous mutant (12.9%), wild-type (6.0%) and reconstituted (9.3%) strains by Day 7	Fiori et al. (2012)
*C. albicans*	N2 Bristol and CB767 [*bli-3(e767)I*] worms from egg stage	Transcription factor Cap1 required for countering reactive oxygen species (ROS)^a^ stress	Cap1 is required for virulence of *C. albicans* in nematode model. Strains lacking CAP1 induced Dar phenotype less frequently with attenuated virulence compared with the wild-type strain. Worms that could not produce ROS due to a mutation in the host oxidase showed early signs of disease and succumbed to an infection with the *cap1Δ/Δ* null mutant	Jain et al. (2013)
*C. albicans*	N2 L4 worms	Magnesium deprivation	20% worm survival after 8 days of treatment compared with 100% mortality in control without treatment	Hans et al. (2019b)
*C. albicans*, *C. dubliniensis*, *C. glabrata*, *C. krusei, C. metapsilosis*, *C. orthopsilosis*, and *C. parapsilosis*	*glp-4; sek-1* L4 worms	Hyphae filamentation, hydrolytic enzymes	*C. albicans* and *C. krusei* were the most virulent with survival rate of 9% by 120 h. At 72 h, *C. parapsilosis* gave a reduced virulence (with no significant difference [*p* = 0.429] from that of *C. glabrata*) with survival rate of 76% compared with 59% and 57% of *C. metapsilosis* and *C. orthopsilosis*, respectively. *C. dubliniensis* gave the least mortality (41%) by the end of the assay. *In vivo* hyphae development was observed only in infected worms with *C. albicans* and *C. krusei*	Ortega-Riveros et al. (2017)
*C. glabrata*, *C. nivariensis*, and *C. bracarensis*	*glp-4; sek-1* L4 worms	None	*C. glabrata* ATCC 90030, NCPF 3203; *C. nivariensis* CECT 11998, CBS 9984; and *C. bracarensis* NCYC 3133, NCYC 3397 gave varying virulence with survival rates of 40.3%/26.5%, 65.4%/45.%1; 72.9%/65.3%, 75%/73%; 89.4%/89.4%, and 97.6%/70.8% in the absence/presence of DMSO (1%), respectively at 120 h	Hernando-Ortiz et al. (2020)
*C. tropicalis*	*glp-4; sek-1* L4 worms	Hyphal filamentation and blastocondia	The mortality rate of the 40 strains from both humans and veterinary ranged from 31% to 98% by 98 h. No significant mortality rate difference (*p* = 0.05) between the human (86.07 ± 3.42) and veterinary (79.8 ± 14.9) strains	Brilhante et al. (2016)
*C*. *parapsilosis* (*sensu stricto*), *C*. *orthopsilosis*, *C*. *metapsilosis*	*glp-4; sek-1* worms	Develop a *C. elegans-Candida parapsilosis* infection model	The 3 *Candida* spp. caused up to 50% mortality of worms ranging from 4 to 6 days. Worms infected by the 3 *Candida* spp. in the liquid assay were susceptible to fluconazole (fluZ) and caspofungin (CAS) and could mount an immune response	Souza et al. (2018)
*C. albicans*	N2 young adult worms	Adhesion/colonization	Increased mortality rate (<10%) compared with the negative control fed with the *Escherichia coli* OP50 (OP50) (˃70%) by Day 9.	Priya and Pandian (2020)
*C. albicans*, *C. glabrata*, and *C. tropicalis*	L4 worms	Colonization and biofilm formation	Decreased survival lifespan of worms infected with *C. albicans* (156 h), *C. glabrata* (180 h), and *C. tropicalis* (252 h) compared with OP50-fed control worms (>312 h).	Muthamil et al. (2018)

a Produced by host.

Moreover, the adoption of a *C. elegans* model for searching and screening of effective bioactive compounds against several species of *Candida* has also received much attention. Effective bioactive compounds from marine habitats (Subramenium et al., 2017; Ganesh Kumar et al., 2019), plant parts (Shu et al., 2016; Pedroso et al., 2019), and other sources (**Table 2**) have been discovered because of their *in vivo* efficacies against several *Candida* species and were simultaneously evaluated for their cytotoxicity in a *C. elegans* model. Compounds such as alizarin, chrysazin, sesquiterpene, and purpurin were discovered to be quite effective in *in vivo* assays with effective doses ranging from 1 to 10 µg/ml (**Table 2**), indicating potential future prospects for antifungal drug research and discovery. Other compounds such as thymol (Shu et al., 2016), coumarin (Xu et al., 2019), and theophylline (Singh et al., 2020), were only effective at high concentrations of 64, 2, and 1.6 mg/ml, respectively (**Table 2**). Most of these compounds were certified as nontoxic at such effective concentrations as they were able to rescue infected nematodes and significantly elongated their lifespan (**Table 2**).

**Table 2:** Evaluation of anticandidal bioactive compounds in the *C. elegans* model.

*Candida* spp.	*C. elegans* host	Effective antifungal compound/agent	Effective concentrations (µg/ml)	Effect	Reference
*C. albicans*	N2 Bristol CF512 *fer-15(b26); fem-1(hc17)* adult worms	Alizarin, chrysazin, and purpurin	≥2	By Day 4, the survival rates of worms in the presence of 2 µg/ml alizarin, chrysazin, purpurin, and fluconazole (fluZ) control were >60%, >50%, >60%, and <50%, respectively. At 1 mg/ml, alizarin had no cytotoxic effect on nematodes whereas chrysazin, purpurin, and fluZ reduced worms survival by >60%, 35%, and >95%, respectively	Manoharan et al. (2017a)
*C. albicans*	N2 young adults	Magnolol and honokiol	16	Both compounds significantly (*p <* 0.0001) protected and increased the lifespan of infected worms compared with infected untreated worms by Day 5. The antifungal compounds also significantly (*p <* 0.01) reduced colonization of *C. albicans* in the nematodes	Sun et al. (2015)
*C. albicans*	Adult worms	Coumarin	2.0 mg/ml	Coumarin at concentrations of 0. 5–2.0 mg/ml significantly (*p <* 0.05) protected infected worms from death. However, coumarin at 2 mg/ml was significantly (*p <* 0.05) toxic to worms	Xu et al. (2019)
*C. albicans*	*glp-4; sek-1* L4 worms	Gallic acid, hexyl gallate, octyl gallate, and dodecyl gallate	1–60	Significant (*p <* 0.05) increased survival rates of worms (13%–33%, 18%–33%, 12%–31%, and 14%–46%) when treated with galic acid, hexyl gallate, octyl gallate, or dodecyl gallate, respectively. Dodecyl gallate was the most effective in protecting worms from *Candidal* infection. However, higher concentrations of these compounds (60 and 120 µg/ml) were toxic to worms	Singulani et al. (2017)
*C. albicans*	N2 worms	Kalopanaxsaponin A (KPA)	8, 16	KPA protected and increased the survival time of worms (5–6 days) compared with the untreated control (4 days). KPA also showed no cytotoxicity on worms at 64 µg/ml for 2 days	Li et al. (2019)
*C. albicans*	*glp-4; sek-1* worms	Chiloscyphenol A (CA)	8, 16	CA significantly (*p <* 0.001) prolonged the survival of infected worms compared with 1% DMSO control. CA at 16 µg/ml prevented hyphae filamentation and maintained worms at their usual curly growth condition. However, CA of ≥32 µg/ml was toxic to worms	Zheng et al. (2018)
*C. albicans*	*glp-4; sek-1* young adult worms	2,6-bis[(E)-(4-pyridyl) methylidene]cyclohexanone (PMC)	8	PMC treatment significantly (*p <* 0.0015) increased the survival rate of infected worms, similar to fluZ treatment at 4 µg/ml	de Sá et al. (2018)
*C. albicans*	AU37 (*sek-1*; *glp-4*) L4 worms	Ebselen	4, 8	Ebselen treatment at 4 and 8 µg/ml significantly (*p <* 0.05) reduced *C. albicans* load in infected worms when compared with the untreated control groups, same as amphotericin B (AmB), fluZ, and flucytosine (fluc) treatments	Thangamani et al. (2017)
*C. albicans*	N2 L4/adult worms	Vanillin (van)	125	Van protected and enhanced the survival of infected worms compared with untreated control within 4 days. Van also had no cytotoxic effects on nematodes by Day 4 of treatment	Venkata et al. (2020)
*C. albicans*	N2 young adults	Floricolin C (FC)	8, 16, 32	FC significantly (*p <* 0.001) enhanced the survival of infected worms at 16 µg/ml giving the highest survival rate compared with the untreated control by Day 6. FC at 64 µg/ml had only little cytotoxic effect on worms within 6 days	Zhang et al. (2018)
*C. albicans*	N2 L4/adult worms	Geraniol (Ger)	135	Ger enhanced the survival of infected nematodes compared to untreated control within 3 days of assay. Ger was also able to reduce persistence of *C. albicans* in worm guts. Furthermore, Ger at 135 µg/ml did not display cytotoxic effect on worms compared to control by Day 3	Singh et al. (2018)
*C. glabrata*, *C. krusei*, *C. tropicalis*, and *C. orthopsilosis*	AU37 late L4 worms	*Cupressus sempervirens* essential oil (EO), *Citrus limon* EO, gallic acid, and *Litsea cubeba* EO	Varied with pathogen and effective compounds	Among the *C. glabrata-*infected worms treated with *C. sempervirens* EO (15.62, 31.25, and 62.5 µg/ml), *C. limon* EO (125, 250, and 500 µg/ml) or gallic acid (15.62, 31.25, and 62.5 µg/ml) for 4 days, only treatment group with *C. sempervirens* EO sustained a higher survival rate of worms (˃60%). *C. krusei*-infected worms treated with *L. cubeba* EO (31.25, 62.5, and 125 µg/ml) or gallic acid (62.5, 125, and 250 µg/ml) did not witness cure from candidiasis. *C. limon* EO treatment (125 and 500 µg/ml) of *C. tropicalis*-infected worms gave 40% and 10%–15% worm survival rate, respectively. While *C. sempervirens* EO treatment (15.62–62.5 µg/ml) of *C. orthopsilosis*-infected worms increased survival rate to 80%–85% Day 4 postinfection. *C. sempervirens* and *L. cubeba* EOs (31.25–125 µg/ml) as well as gallic acid (15.62–250 µg/ml) were not toxic to worms compared with untreated control. Additionally, *C. limon* EO at 125 µg/ml was not toxic to worms but became significant toxic at higher concentrations of 250 µg/ml (*p <* 0.05) and 500 µg/ml (*p <* 0.0001) compared with untreated control	Pedroso et al. (2019)
*C. albicans*	N2 L4/young adult worms	Monoterpenoid perillyl alcohol (PA)	175 and 350	PA enhanced and prolonged infected nematodes with survival rates of 80% and 75% at 175 and 350 µg/ml, respectively, compared with untreated control with 16% survival by Day 7 postinfection. The persistence of *C. albicans* in the intestines of worms was reduced by PA. PA was also not toxic to *C. elegans* at 350 µg/ml after 7 days of incubation	Ansari et al. (2018)
*C. albicans*	*glp-4; sek-1* worms	Solasodine-3-*O*-*β*-d-glucopyranoside (SG)	≥8	SG significantly (*p <* 0.0001) protected and prolonged the lifespan of infected *C. elegans* compared with the 1% DMSO control, inhibiting the hyphal filamentation of *C. albicans* in infected worms by Day 6 of postinfection. Moreover, SG was not toxic to worms at 64 µg/ml in 2 days of incubation	Li et al. (2015)
*C. albicans*	N2 L4/young adult worms	Theophylline (THP)	1,600	THP gave over 50% more survival rate of infected worms than the untreated infected control after 6 days postinfection. THP was able to drastically lower the persistence of *C. albicans* in nematode gut. Additionally, THP did not show any toxicity at 1.6 mg/ml compared with untreated control for 6 days of treatment	Singh et al. (2020)
*C. albicans*	N2 and several mutant^a^ worms	Thymol	64 mg/ml	Thymol significantly (*p <* 0.01) increased the survival rate and mean lifespan (10.5 ± 0.4 days) of infected *C. elegans* compared with untreated infected worms (6.1 ± 0.5 days) within 10 days postinfection. Thymol elicited important immunomodulatory response of *C. elegans* against *C. albicans* thus significantly (*p <* 0.01) reduced fungal burden in treated infected worms compared with untreated control	Shu et al. (2016)
*C. albicans*	Young adult worms	Sesquiterpene compound	≥10	Sesquiterpene compound prolonged the lifespan of infected worms with >70% survival rate up to 20 µg/ml treatment but became toxic at higher concentration of 50 µg/ml compared with untreated control	Ganesh Kumar et al. (2019)

a KU25/pmk-1(km25) IV, AU1/sek-1(ag1) X, FK171/mek-1(ks54) X, AU3/nsy-1(ag3) II, and DA1750/adEx1750[PMK-1::GFP+rol 6(su1006)].

The drug resistance threat of *Candida* species, similar to most other pathogens, is constantly increasing, leading to increased incidences of mortality and morbidity (Sanguinetti et al., 2015; Popp et al., 2017; Popp et al., 2019; Prasad et al., 2019). *C. elegans* has also proven to be an effective *in vivo* model for studying the infection of several azole-resistant *C. albicans* (Chang et al., 2015; Sun et al., 2018) and *C. auris* (Eldesouky et al., 2018a; Eldesouky et al., 2018b) strains. Studies have demonstrated the *in vivo* efficacy of some bioactive compounds applied singly or in combination with initially resistant antifungal drugs in the treatment of infected nematodes (**Table 3**).

**Table 3:** Evaluation of effective agents against drug-resistant *Candida* species in a *C. elegans* model.

*Candida* spp.	*C. elegans* host	Kind of drug resistance and MIC	Antifungal compound/agent	Time of preinfection (min)	Effect	Reference
*C. albicans*	AU37 L4 worms	fluZ	2-(5,7-Dibromoquinolin-8-yl)oxy)-*N*′-(4-nitrobenzylidene) acetohydrazide (4b)	90	Compound 4b exhibited broad-spectrum antifungal activity towards species pf *Candida*, *Cryptococcus*, and *Aspergillus* at a concentration of 0.5 µg/ml, as well as enhanced survival of *C. elegans* infected with fluz-resistant *C. albicans*. This compound targets metal ion homeostasis	Elghazawy et al. (2017) and Mohammad et al. (2018)
*C. albicans*	N2 worms	fluZ 256 µg/ml	Caffeic acid phenethyl ester (CAPE) and fluZ	120	CAPE plus fluZ synergistically increased the survival rate of infected worms significantly compared with single treatment with either CAPE or fluZ. CAPE plus fluZ also significantly (*p <* 0.01) reduced *C. albicans* burden in nematode intestines compared with just CAPE, fluZ, or the untreated control (all at 2 µg/ml)	Sun et al. (2018)
*C. albicans* and *C. auris*	AU37 L4 worms	fluZ >64 µg/ml	Phenylthiazole small molecule (compound 1)	90	Compound 1 (at 5 and 10 µg/ml) enhanced the survival of *C. albicans*-infected nematodes, giving >70% survival rate (just like 5 µg/ml of 5-fluorocytosine control) by Day 3 postinfection compared with 0% of untreated infected worms. Similarly, Compound 1 (at 10 µg/ml) prolonged *C. auris*-infected worms giving ~70% survival by Day 4 compared with 0% of untreated infected worms	Mohammad et al. (2019)
*C. albicans*	*glp-4; sek-1* worms	fluZ >128 µg/ml	Pyridoxatin (PYR)	120	PYR rescued and prolonged infected nematodes in a dose-dependent manner with 4 µg/ml giving ~50% survival rate after 5 days of treatment	Chang et al. (2015)
*C. albicans*	AU37 L4 worms	fluZ >64 µg/ml; itraconazole (itZ) and voZ >16 µg/ml	Sulfa drugs^a^ + fluZ	180	Sulfa (10 × MIC^b^) and fluZ (10 µg/ml) combinations gave a significant (*p* < 0.05) reduction of *C. albicans* burden in infected worms (which is comparable with 5-fluorocytosine control) after 24 h treatment compared with fluZ and the DMSO-untreated controls. There was no significant difference among the activities of the 4 sulfa with fluZ combinations	Eldesouky et al. (2018b)
*C. auris*	AU37 L4 worms	Azole resistant; fluZ >128 µg/ml; voZ = 16 µg/ml; itZ = 2 µg/ml	Sulfamethoxazole + voZ	30	The combination of sulfamethoxazole (128 µg/ml) with voZ (0.5 µg/ml) prolonged the life of infected worms by ~70% as against only sulfamethoxazole, voZ, or untreated control which could not keep worms alive till Day 5	Eldesouky et al. (2018a)

MIC, minimum inhibition concentration. ^a^Sulfamethoxazole (SMX), sulfadoxine (SDX), sulfadimethoxine (SDM), or sulfamethoxypyridazine (SMP). ^b^MICs of SMX, SDX, and SMP = 512 µg/ml, while MIC of SDM = 1,024 µg/ml.

Compounds such as 2-(5,7-dibromoquinolin-8-yl)oxy)-*N*′-(4-nitrobenzylidene) acetohydrazide (Elghazawy et al., 2017; Mohammad et al., 2018) and phenylthiazole small molecules (Mohammad et al., 2019) are among the recently demonstrated effective compounds with good outcomes in nematode candidiasis (with effective dose concentrations of ≥4 and ≥5 µg/ml, respectively) against fluZ-resistant *C. albicans* and/or *C. auris* (**Table 3**). The combination of caffeic acid phenethyl ester (CAPE) and fluZ (Sun et al., 2018) as well as the sulfamethoxazole and voriconazole (voZ) combination (Eldesouky et al., 2018a) effectively rescued *C. elegans* worms infected by azole-resistant *C. albicans* and *C. auris*, respectively (**Table 3**).

The search for alternative treatment drugs with new inhibition mechanisms against pathogenic fungi such as *C. albicans* is a pressing need. Obtaining effective compounds that may not necessarily have a direct effect on *Candida* planktonic cells but affect critical virulence factors has recently been made possible by evaluating the efficacy of the compounds in a *C. elegans* infection model (Graham et al., 2017; Subramenium et al., 2017; Manoharan et al., 2018) (**Table 4**).

**Table 4:** *C. elegans* model demonstrating alternative inhibition mechanisms against *Candida* species.

*Candida* spp.	*C. elegans* host	Effective antifungal agent	Effective concentrations (µg/ml)	Effect		Reference
*C. albicans*	N2 Bristol *CF512 fer-15*; *fem-1* adult worms	7-Benzyloxyindole	0.05 mM	7-Benzyloxyindole gave nematode survival rate of >40% while the positive control (fluZ) gave >60% by Day 4, both showed significant (*p <* 0.05) increase of survival rates compared with the untreated control (8%). 7-Benzyloxyindole at 0.1 mM showed mild toxicity on worms with 22% survival rate compared with 55% survival by fluZ. 7-Benzyloxyindole protected infected worms by preventing hyphal filamentation through downregulation of important hyphae-specific and biofilm-related genes		Manoharan et al. (2018)
*C. albicans*	*glp-4; sek-1* young adult worms	*Enterococcus faecalis* bacteriocin (EntV)	0.1 nM	Synthetic EntV (sEntV^68^) completely abrogated the virulence of *C. albicans* in infected worms, giving them lifespan similar to control worms fed with nematode food *E. coli* OP50. sEntV^68^ had no effect on the viability of *C. albicans* but protected the nematode by preventing hyphal morphogenesis.		Graham et al. (2017)
*C. albicans*	*fer*-*15*; *fem*-*1* adult worms	Cascarilla bark oil, α-longipinene, and linalool	≥0.001%	Separate treatments with cascarilla bark oil, α-longipinene, and linalool resulted in a significant (*p <* 0.05) increase in survival rate (>90%) of infected nematodes just like fluZ treatment (all at 0.01%) compared with the negative control (<5%) by Day 4. These antifungal compounds only became toxic at >0.5% (v/v) to the worms. Cascarilla bark oil, α-longipinene, and linalool protected infected worms by preventing hyphal filamentation but no direct effect on *C. albicans* planktonic cells		Manoharan et al. (2017c)
*C. albicans*	*glp-4; sek-1* adult worms	Loureirin A (Lou A)	40	Lou A significantly (*p <* 0.05) protected infected nematodes compared with the DMSO control in 144 h. More so, Lou A did not display any cytotoxic activity against the worms at 160 µg/ml. At effective *in vivo* concentration of 40 µg/ml, Lou A did not inhibit the growth of *C. albicans* but suppressed virulence trait such as adhesion, colonization, and hyphal filamentation		Lin et al. (2019)
*C. albicans*	N2 young adult worms	Piperine	≥BIC (32)	Piperine treatment helped worms to combat infection in a dose-dependent manner leading to a significant (*p <* 0.05) reduction in *C. albicans* load. Piperine did not result in cytotoxity at sub-BIC, BIC, and 2 × BIC in worms. Piperine *in vivo* efficacy was mainly through hindering *C. albicans* colonization in nematode intestine by downregulating some important hyphae-specific genes but not affecting the growth and metabolism of the pathogen		Priya and Pandian (2020)
*C. albicans*, *C. glabrata*, and *C. tropicalis*	L4 worms	Quinic acid and undecanoic acid (QA-UDA)	BIC^a^ (100)	QA-UDA at BIC increased the survival rates of worms infected by *C. albicans*, *C. glabrata*, and *C. tropicalis* to 216, 384, and 348 h compared with 156, 180, and 252 h of untreated infected worms, respectively. QA-UDA reduced *in vivo* biofilm formation and colonization of yeast pathogens in worms	Muthamil et al. (2018)	
*C. albicans*	*fer-15*; *fem-1* adult worms	Camphor and fenchyl alcohol	0.01%	Treatment of infected worms with camphor and fenchyl alcohol significantly (*p <* 0.05) increased the survival rates of infected worms to >70% and >50%, respectively, compared with 5% untreated control. These compounds had no effect on worm survival and viability at concentrations of 0.05% and 0.1% in 4 days, but they became significantly toxic (*p <* 0.05) at 0.5%. Camphor and fenchyl alcohol at BIC (approximately 50 times the MIC) had effect on *C. albicans* biofilm and hyphal filamentation but not on the planktonic cells	Manoharan et al. (2017b)	
*C. albicans*	L4 worms	5-Hydroxymethyl-2-furaldehyde (5HM2F)	MBIC (400)	Increased survival time of infected worms when treated with 5HM2F (120 h) compared with 96 h of control group. 5HM2F displayed no cytotoxic effect on worms by120 h. 5HM2F below 500 µg/ml does not have antifungal effect on *C. albicans* except on some virulence factors such as biofilm formation, morphological transition, and production of secreted hydrolases	Subramenium et al. (2017)	

a BICs for C. albicans, C. glabrata, and C. tropicalis in combination with QA/UDA were 100/5, 100/10, and 200/20 µg/ml, respectively. BIC, biofilm inhibition concentration; MBIC, minimum biofilm inhibitory concentration.

Remarkably, some compounds such as loureirin A (Lin et al., 2019), camphor, and fenchyl alcohol (Manoharan et al., 2017b) are effective compounds protecting infected worms at concentration doses less than the *in vitro* MICs (**Table 4**). Cascarilla bark oil, α-longipinene, linalool (Manoharan et al., 2018), and *Enterococcus faecalis* bacteriocin (EntV) (Graham et al., 2017) were reported to be quite potent in rescuing infected worms at low effective concentration doses, such as ≥0.001% for cascarilla bark oil, α-longipinene and linalool and 0.1 nM for EntV (**Table 4**).

These compounds usually rescue infected nematodes through other pathways such as direct effects on cardinal virulence factors and/or by stimulating/enhancing the immune responses of the host against pathogens (Okoli et al., 2009; Peterson and Pukkila-Worley, 2018; Ahamefule et al., 2020b). Such compounds may only be screened and identified through *in vivo* assays since they usually show little or no antimicrobial activities in *in vitro* assays. The adoption of simple *in vivo* models such as *C. elegans* significantly supports the screening and identification of more such compounds, which may expand the narrative of the usual antifungal therapies that primarily address direct effects on causative pathogens.

The application of live biotherapeutic products (LBPs) consisting mainly of probiotics is another alternative approach for the treatment of nematode candidiasis. Such alternative therapy is an interesting and promising option since pathogenic fungi are currently developing resistance to the few clinically available antifungal drugs (Sanguinetti et al., 2015; Prasad et al., 2019). Several species of *Lactobacillus* such as *L. rhamnosus* (Poupet et al., 2019a; Poupet et al., 2019b) and *L. paracasei* (de Barros et al., 2018) as well as probiotic yeasts—*Saccharomyces cerevisiae* and *Issatchenkia occidentalis* (Kunyeit et al., 2019)—have demonstrated efficient rescue of worms infected with a number of *Candida* species. These therapeutic microorganisms drastically reduced the burden of the pathogens in the *C. elegans* intestine approximately 2 to 4 h postinfection treatment (**Table 5**).

**Table 5:** Application of live biotherapeutic products (LBP) to nematode candidiasis.

*Candida* sp.	*C. elegans* host	LBP	Time of preinfection (h)	Effective concentartions/time	Effect	Reference
*C. albicans*	N2 L4/young adult worms	*Lactobacillus rhamnosus* Lcr35^®^ (Lcr35)	2	2 and 4 h	High significant (*p* < 2 × 10^−16^) increase by Lcr35 treatment in mean lifespan (from 4 to 13 days) of worms sequentially infected with *C. albicans* compared with untreated control. However, increasing Lcr35 treatment to 6 and 24 h led to a significant decrease in mean lifespan of worms compared with 4 h treatment	Poupet et al. (2019a)
*C. albicans*	*glp-4; sek-1* young adult worms	*Lactobacillus paracasei* 28.4	2 and 4	2 and 4 h	*L. paracasei* significantly (*p* = 0.0001) attenuated the death rate of infected worms (with 29% increase in survival rate) compared with untreated infected worms by Day 10 of assay	de Barros et al. (2018)
*C. albicans*	N2 L4/young adult worms	*Lactobacillus rhamnosus* Lcr35^®^ (Lcr35)	2	2 and 4 h	2 h Lcr35 treatment gave a significant (*p* < 2 × 10^−16^) increase in the mean lifespan of infected worms (from 3 to 11 days) compared with untreated infected worms. Lcr35 prevented hyphae filaments in infected worms although it could not totally eradicate pathogens from the intestine of worms. Feeding nematodes with Lcr35 alone significantly increased the mean lifespan of worms compared with *E. coli* OP50. Increasing Lcr35 treatment time beyond 4 h gave a significant drop in worm survival	Poupet et al. (2019b)
*C. tropicalis*, *C. krusei*, *C. parapsilosis*, and *C. glabrata*	L3 and L4 worms	Probiotic yeasts: *Saccharomyces cerevisiae* (strain KTP) and *Issatchenkia occidentalis* (strain ApC)	48	10^6^ cells/20 µl	Significant increase in lifespan (by 5–6 days) of worms coinfected by any of yeast pathogens—*C. tropicalis* (*p ≤* 0.0001), *C. krusei* (*p <* 0.0012), and *C. parapsilosis* (*p <* 0.0001)—and the probiotic yeasts compared with their controls without treatments. However, such increase lifespan was not recorded for *C. glabrata* infection. The probiotics treatments significantly (*p <* 0.05) reduced pathogen colonization in the gut of nematodes with no CFU recovered at Day 5 after postinfection probiotics treatments	Kunyeit et al. (2019)

CFU, colony forming unit (in CFU/ml).

The efficacy of these LBPs in reducing and/or eliminating fungal burden implies the future potential of LBPs in addressing the fungal menace. The demonstrated significant increase (*p* < 2 × 10^−16^) in worm mean lifespan (Poupet et al., 2019a; Poupet et al., 2019b) is so high that it has not been reported in any potent bioactive compounds or even established antifungal drugs. The fact that most of these LBPs are already established probiotics is yet another important parameter that would advance future research beyond nematode models.

The *in vivo* efficacy of known antifungal drugs and a number of repurposed drugs have also been applied in the treatment of nematode candidiasis. Several azoles (Souza et al., 2018; Hernando-Ortiz et al., 2020), echinocandins (Souza et al., 2018), polyenes—particularly amphotericin B (Hernando-Ortiz et al., 2020), and β-lactam antibiotics (in combination with vancomycin) (De Aguiar Cordeiro et al., 2018) have been evaluated for their *in vivo* efficacy at varying effective concentrations in rescuing worms infected with *Candida* species (**Table 6**). Synthesized azole drugs, such as 1-(4-cyclopropyl-1H-1,2,3-triazol-1-yl)-2-(2,4-difluorophenyl)-3-(1H-1,2,4-triazol-1-yl) propan-2-ol, have also been evaluated for both efficacy and cytotoxicity in a *C. elegans* model (Chen et al., 2017).

**Table 6:** *In vivo* activities of known and repurposed drugs against candidiasis in *C. elegans* models.

*C. elegans* sp.	*C. elegans* host	Antifungal compounds	Effective concentrations (µg/ml)	Effect	Reference
*C. glabrata*, *C. nivariensis*, and *C. bracarensis*	*glp-4; sek-1* L4 worms	Micafugin (MCF), CAS, and fluZ were prepared in water, AmB, VoZ, posaconazole (PoZ), and anidulafungin (AND) in 1% DMSO	Varying	MCF (4 µg/ml), CAS (4 µg/ml), AmB (1 µg/ml), and voZ (2 µg/ml), poZ (2 µg/ml) rescued infected worms with *C. glabrata* ATCC 90030 with survival rates of 90.6, 89.6, 82.4, 82.1, and 81.5%, respectively, by 120 h; higher similar rescues—96.8%, 94.6%, 91.8%, 85.2%, 83.8%, and 83.7%—were achieved for infected worms with *C. glabrata*	Hernando-Ortiz et al. (2020)
*C*. *parapsilosis* (*sensu stricto*), *C*. *rthopsilosis*, *C*. *etapsilosis*	*glp-4; sek-1* worms	fluZ and CAS	≥0.5 × MIC (fluZ MIC = 1.0; CAS MIC = 0.5)	Worm survival rates were dependent on the drug doses. Significant (*p <* 0.001) increase in survival of infected worms when treated with fluZ (≥57%) and CAS (69% and 74%) at 1 × MIC and 2 × MIC, respectively	Souza et al. (2018)
*C. albicans*, *C. parapsilosis*, *C. krusei*, and *C. tropicalis*	L4 worms	Cefepime (cef), imipenem (imi), meropenem (mer), amoxicillin (amo), and vancomycin (van)	PP and 2 × PP PP of cef, imi, mer, amo, and van = 126; 33, 33, 4, and 15, respectively	Amo treatment significantly (*p <* 0.05) increased the virulence of *C. krusei* and *C. tropicalis* on the nematodes (in separate infections) at PP and 2 × PP. However, the virulence of *C. albicans*, *C. krusei*, *C. parapsilosis*, and *C. tropicalis* were not altered by the other tested antibiotics	De Aguiar Cordeiro et al. (2018)
*C. albicans*	*glp-4; sek-1* adult worms	1-(4-Cyclopropyl-1H-1,2,3-triazol-1-yl)-2-(2,4-difluorophenyl)-3-(1H-1,2,4-triazol-1-yl) propan-2-ol (7l)	16	7l significantly (*p <*0.05) prolonged and sustained infected worms, giving 70% survival rate compared with 60% recorded with 32 µg/ml of fluZ control	Chen et al. (2017)
*C. albicans*	N2 L4/young adult worms	Theophylline (THP)^a^	1,600	THP gave over 50% more survival rate than the untreated infected control after 6 days postinfection. THP was able to drastically lower the persistence of pathogen in nematode gut. Additionally, THP did not show any toxicity at 1.6 mg/ml compared with untreated control by Day 6	Singh et al. (2020)
*C. albicans*, *C. glabrata*, and *C. auris*	AU37 L4 worms	Pitavastatin (Pit)^a^ plus fluZ	Varying^b^	Pit plus fluZ displayed broad spectrum activity with varying outcomes depending on fluZ concentrations, and significantly reduced *C. albicans*, *C. glabrata*, and *C. auris* burden by ~82%–96%, ~84%–93% and 14%–92% compared with 233 ± 21, 344 ± 19, and 250 ± 25 CFU/ml of untreated controls, respectively	Eldesouky et al. (2020b)

MIC, minimum inhibition concentration; PP, peak plasma concentration. ^a^Repurposed drug. ^b^Pit = 0.5 × MIC; fluZ = 2, 8, and 32 µg/ml.

Given that decades of searching for new antifungal agents have not truly resulted in new antifungal drugs, drug repurposing is a less expensive and welcome research prospect. The *C. elegans* infection model for evaluating the efficacy of repurposed drugs on candidiasis has attracted attention (Eldesouky et al., 2020b; Singh et al., 2020) (**Table 6**) due to the advantages of saving extensive time, cumbersome labor, and enormous cost of searching and obtaining new antifungal drugs.

### *C. elegans* and Pathogenic Molds

The deadly opportunistic mold pathogen, *A. fumigatus*, ranks as the number 1 aetiological agent for aspergilloses in immunocompromised patients (Snelders et al., 2009; Fang and Latgé, 2018; Geißel et al., 2018) with an almost 100% mortality rate in some groups of patients (Darling and Milder, 2018; Geißel et al., 2018; Linder et al., 2019). This pathogen had not been well studied in *C. elegans* until recently. Okoli and Bignell (2015) were the first to demonstrate the possibility of adopting *C. elegans* for *A. fumigatus* infection. They set up the nematode model to study the pathogenicity of the clinical strain *A. fumigatus* Af293 for 72 h postinfection after an initial preinfection of 12 h. We recently reported a breakthrough in overcoming some of the challenges usually encountered in the *C. elegans-*mold infection system, one of which is removing spores that were not ingested by worms through a hand-made filter with a membrane-attached-on-tube. We were able to develop a stable and consistent *C. elegans* model for evaluating the virulence of *A. fumigatus* mutant strains that had previously been studied in other established models, including mice and insects. We also successfully demonstrated the possibility of *in vivo* testing of antifungal agents on nematode aspergillosis using the established model (Ahamefule et al., 2020a).

The established *C. elegans-A. fumigatus* model clearly demonstrated the progression of aspergillosis infection in nematodes using the *A. fumigatus* fluorescence strain, Af293-dsRed, showing that hyphal filamentation could actually emanate from any part of the infected worms against the previously reported concept of mainly the tail region (Okoli and Bignell, 2015; Ahamefule et al., 2020a). Our worm model was able to identify important virulence factors of *A. fumigatus* such as α-(1,3)-glucan synthase, melanin pigmentation, iron transporter, Zn2Cys6-type transcription factor, and mitochondrial thiamine pyrophosphate transporter, as mutant strains without these components (triple *agsΔ*, *pksPΔ*, *ΔmrsA*, *ΔleuB*, and *ΔtptA*, respectively), all of which gave significantly attenuated virulence compared with the *A. fumigatus* parent strain KU80Δ. These reduced virulence patterns obtained by our *C. elegans* model were similar to previously reported attenuated virulence patterns of these *A. fumigatus* mutants in both vertebrate and insect models. The nematode model was also demonstrated to be an easy *in vivo* system to evaluate antifungal drug efficacy thus presenting the model as a desired platform for screening antifungal agents against *A. fumigatus* in the future (Ahamefule et al., 2020a).

## Challenges of *C. elegans* Applications in Modeling Pathogenic Mold

One of the biggest challenges usually encountered in the applications of the *C. elegans* model for filamentous fungal infection is the difficulty in infecting the worms through conidia. Worms usually avoid eating conidia unless they starve with no other option (Okoli and Bignell, 2015). This avoidance is unlike the case of dimorphic fungal and bacterial pathogens, where infection is never much of a problem as worms easily feed on the cells of these pathogens when they replace or are mixed up with nematode choice food (*E. coli* OP50 or HB101) (Breger et al., 2007; Johnson et al., 2009; Kirienko et al., 2013; Okoli and Bignell, 2015).

Giving the worms more time to starve and more access to the conidia (placed at four cardinal points) for ingestion is very important for establishing mold preinfection assays. Okoli and Bignell (2015) adopted a 12-h preinfection technique, while we modified to 16 h (Ahamefule et al., 2020a). The fact is that worms must be given such ample time to “force” them to ingest the mold conidia in a preinfection system since coinfection approach (which is usually adopted for most dimorphic fungi modeling) cannot work well for mold pathogens (Okoli et al., 2009; Okoli and Bignell, 2015; Ahamefule et al., 2020a). As conidia germinate very fast even before the worms have ingested enough spores in killing assay medium, a relatively less nutritious medium was adopted for pre-infection assay to avoid the quick growth and flooding of hyphal filaments in the rich killing assay medium (brain heart infusion medium); otherwise later experimental procedures will be severely limited (Okoli and Bignell, 2015; Ahamefule et al., 2020a).

**Figure 1:**
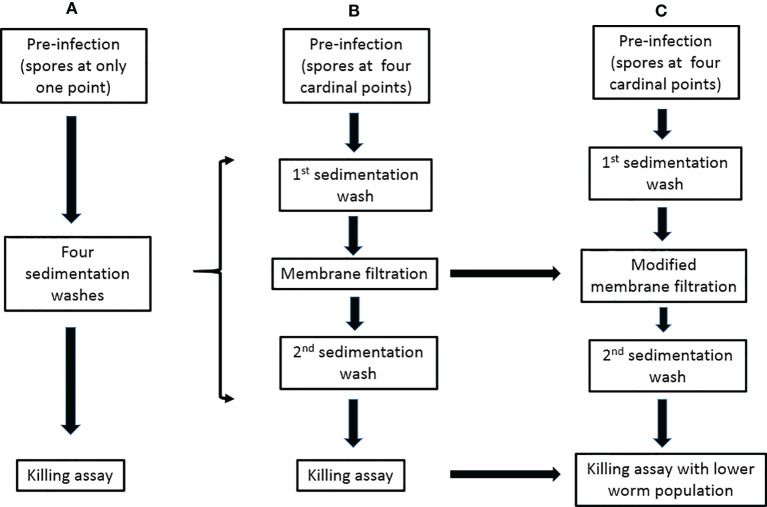
Modifications of the preinfection to killing assays for the *C. elegans*-mold infection model. **(A)** The previously described procedure (Okoli and Bignell, 2015). **(B)** The procedure in our publication (Ahamefule et al., 2020a). **(C)** Our proposed modifications.

Another challenging aspect in setting up the *C. elegans-*mold model is the separation of noningested conidia from worms after pre-infection stage. Failure at this stage leads to the germination of unseparated spores in killing or antifungal screening media thus obstructing experimental progress. Although our designed membrane-attached-on-tube filter (with a 35-µm pore diameter) was able to remove a great deal of noningested conidia, the separation was not 100% efficient. Modifying the membrane pore size to an appropriate diameter should help improve the filtration efficiency by allowing faster and better removal of conidia while keeping the preinfected L4/young adult worms (**Figure 1**). Even though the separation efficiency of noningested spores becomes 100% or close to it, hyphae growth in killing medium would still not be completely eliminated, particularly if the experiment is scheduled to go beyond 72 h postinfection. This is because we have discovered that some conidia could be egested out of the nematode intestine into the killing medium and still retain their viability of germinating to hyphae, which is a big challenge to tackle and severely affect the experiment.

Hyphal filamentation usually occurs in infected worms. Unlike most studied dimorphic fungi whose external hyphae protrude when worms were already dead (and could therefore be easily transferred), numerous worms infected with filamentous fungi such as *A. fumigatus* (Ahamefule et al., 2020a), *A. flavus*, and some strains of *Penicillium* (that we have studied in our laboratory), were discovered to still be alive with protruded hyphae. This makes these worms stuck to the killing assay plates and therefore difficult to remove (Ahamefule et al., 2020a). Such filamentation usually becomes profuse, growing and spreading very fast and may eventually obstruct visibility and affect the experimental results. Regulating the number of immunocompromised worms in killing assays, especially for highly virulent pathogenic molds, is an option to ameliorate this menace (**Figure 1**).

## Conclusions

The tremendous health hazards of pathogenic fungi cannot be overemphasized. Better understanding of *in vivo* pathogeneses and identification of virulence factors are urgent and imperative to fight against these fungi. Screening, identifying and repurposing effective compounds/drugs against them as well as obtaining and optimizing effective treatment alternatives are desirable at this time. Therefore, developing, optimizing and applying better modelling organisms such as *C. elegans* is meaningful not only for dimorphic fungi but also for mold pathogens. Our review of the breakthrough applications of *C. elegans* for dimorphic fungi studies and progress/modifications of the *C. elegans-*mold infection model will provide a reference for studying fungal infections and developing antifungal agents.

## Author Contributions

CA and BE wrote the initial manuscript. JO, AM, AI, BW, CJ, and WF revised the manuscript. WF supervised the manuscript. All authors have read and agreed to the published version of the manuscript.

## Funding

This work was supported by National Natural Science Foundation of China (31960032, 32071279), Guangxi Natural Science Foundation (2020GXNSFDA238008) to WF, Research Start-up Funding of Guangxi Academy of Sciences (2017YJJ026) to BW, and Bagui Scholar Program Fund (2016A24) of Guangxi Zhuang Autonomous Region to CJ.

## Conflict of Interest

The authors declare that the research was conducted in the absence of any commercial or financial relationships that could be construed as a potential conflict of interest.

## Publisher’s Note

All claims expressed in this article are solely those of the authors and do not necessarily represent those of their affiliated organizations, or those of the publisher, the editors and the reviewers. Any product that may be evaluated in this article, or claim that may be made by its manufacturer, is not guaranteed or endorsed by the publisher.
